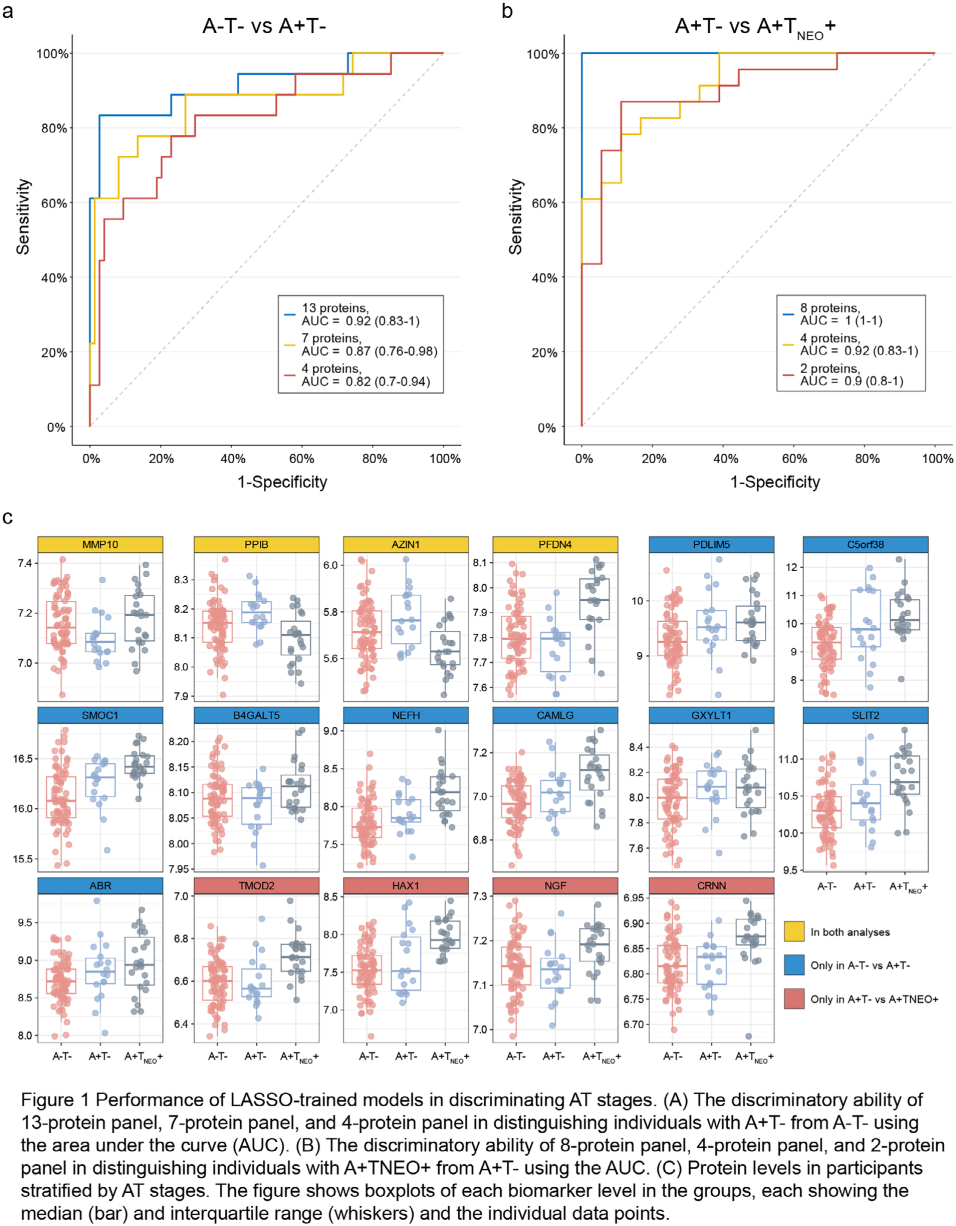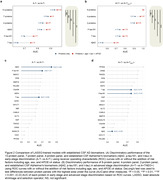# Cerebrospinal fluid proteomics identifies biomarkers for discriminating amyloid PET and tau PET stages

**DOI:** 10.1002/alz.085720

**Published:** 2025-01-09

**Authors:** Zhibo Wang, Yuhan Chen, Jianping Jia

**Affiliations:** ^1^ Xuanwu Hospital, Capital Medical University, Beijing, Beijing China; ^2^ Hebei North University, Zhangjiakou, Hebei China; ^3^ Innovation Center for Neurological Disorders, Xuanwu Hospital, Capital Medical University, Beijing, Beijing China

## Abstract

**Background:**

Staging Alzheimer's disease pathology is critical for therapeutic trials and prognosis. Disease staging with amyloid and tau PET provides high accuracy, but this would be more practical with fluid biomarkers. However, current fluid biomarkers are deficient in tracking specific disease stages, especially in advanced stages.

**Method:**

Participants (N=115) were enrolled from the Alzheimer’s Disease Neuroimaging Initiative who had available CSF proteomics and amyloid and tau PET. The proteomic analysis involved approximately 6000 unique CSF proteins measured by SomaScan 7K. A least absolute shrinkage and selection operator (LASSO) regression combined with machine learning approach was employed to develop proteomic predictive models for amyloid PET and tau PET stages. Finally, we compared the model's performance with established CSF Alzheimer's biomarkers (amyloid‐β [Aβ]42, phosphorylated tau [p‐tau]181, and total tau [t‐tau]).

**Result:**

We identified 38 proteins associated with amyloid PET and 128 proteins with tau PET in the amyloid PET‐positive participants. These proteins are primarily related to glucose metabolism and neurotrophic function. Through machine learning, 17 proteins were selected for the panel to differentiate amyloid and tau PET stages. Of these, 9 proteins were specific for distinguishing between amyloid PET‐negative and tau PET‐negative (A‐T‐) and amyloid PET‐positive and tau PET‐positive (A+T‐) (e.g., SMOC1, NEFH), 4 for discriminating A+T‐ and amyloid PET‐positive and tau PET‐positive in the temporal neocortex (A+TNEO+) (e.g., TMOD2, CRNN), with 4 common to both categories (e.g., MMP10, PPIB). We found that a maximum of 8 and a minimum of 2 proteins effectively differentiated A+TNEO+ from A+T‐ (area under curve range: 0.9‐1), and a maximum of 13 and a minimum of 4 proteins discriminated A+T‐ from A‐T‐ (area under curve range: 0.82‐0.92) (Figure 1). The discriminative performance of these protein panels was significantly superior to the established CSF Alzheimer's biomarkers, with or without the addition of risk factors in the models (Figure 2), which we validated using autopsy data.

**Conclusion:**

In conclusion, assessing a limited set of proteins can accurately determine distinct pathological stages of Alzheimer's disease, addressing the limitations of current fluid biomarkers in identifying specific disease stages.